# Reflections on the I-squared index for measuring inconsistency in meta-analysis

**DOI:** 10.1017/rsm.2025.10062

**Published:** 2025-12-29

**Authors:** Julian P. T. Higgins, José A. López-López

**Affiliations:** 1 Population Health Sciences, Bristol Medical School, University of Bristol, Bristol, UK; 2 National Institute for Health and Care Research Applied Research Collaboration West at University Hospitals Bristol and Weston NHS Foundation Trust, Bristol, UK; 3 Department of Basic Psychology and Methodology, University of Murcia, Murcia, Spain; 4 Murcian Institute of Biomedical Research, IMIB-Arrixaca, Murcia, Spain

**Keywords:** combining information, evidence synthesis, heterogeneity, I-squared, inconsistency, meta-analysis

## Abstract

The I-squared index was proposed in 2002 as a measure to help understand the consistency of study results in a meta-analysis. It was developed to overcome some of the limitations of existing measures, principally the chi-squared test for heterogeneity and the between-study variance as estimated in a random-effects meta-analysis. I-squared measures approximately the proportion of total variability in results that is due to true heterogeneity rather than random error; it is also conveniently interpreted as a measure of inconsistency in the results of the studies. The index has become extremely widely used, although it is often misinterpreted as an absolute measure of the amount of heterogeneity, which it is not. Here, we discuss the I-squared index and the different ways it can be defined, computed, and interpreted. We introduce a new interpretation of I-squared as a weighted sum of squares, which we propose may be helpful when setting up simulation studies. We discuss some of the extensions and repurposes that have been proposed for I-squared and offer some recommendations on the appropriate use of the index in practice.

## Highlights

### What is already known?

- The I-squared index is widely used in relation to the identification or description of heterogeneity in meta-analysis.
- The role of I-squared has been questioned, partly because it is often misinterpreted: It measures inconsistency in findings, which is not the same as variability in effect sizes.

### What is new?

- We provide a technical overview and our reflections on the I-squared index, including its definition, computation, and interpretation, and we offer a new definition that might be particularly useful when designing simulation studies.

### Potential impact for RSM readers

- Given the routine use of the I-squared index in diverse research fields, it is important that users have a proper understanding of what it can, and cannot, portray.

## 1 Introduction

Meta-analysis is the statistical integration of results from a set of studies considered to be sufficiently similar for the combined estimate to be meaningful. If the studies are very similar such that they can be assumed to have addressed the same research question, their results might be expected to differ only because of sampling error. If the studies are all very large, the variation among their results would then be small. However, in practice, studies tend to differ in aspects such as the sample characteristics, ways in which treatments or exposures are defined or measured, and ways in which outcomes are measured. As a result, some degree of additional variation among the results is to be expected.^1^^,^ ^2^ Such variation beyond what can be attributed to chance alone is usually called *statistical heterogeneity* or (in statistical texts) simply *heterogeneity*. It may be measured using, for example, the standard deviation of true effects across studies on the scale in which the effect size is measured (e.g., as a mean, a mean difference, or (log) odds ratio).

The 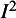 index is a descriptive statistic that is used in relation to heterogeneity among studies in a meta-analysis. It aims to quantify *inconsistency*, a term we use for a construct that is distinct from (statistical) heterogeneity. Inconsistency relates to the extent to which the statistical findings of the studies disagree with each other after taking into account sampling error. Interpretation of inconsistency measures such as the 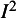 index does not depend on the scale on which the result is measured. As such, they cannot be used to infer the actual (or absolute) amount of heterogeneity in the effect sizes across studies. The 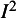 index is typically expressed as a percentage, with a value of 0% indicating that the only source of variability among the results reported in the primary studies is sampling error (so there is no heterogeneity) and values approaching 100% indicating that nearly all variability is due to heterogeneity among the studies rather than sampling error.

The index was introduced under the nomenclature 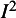 by Higgins and Thompson (HT) in 2002, motivated by a desire to find an alternative to either the standard 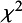 test for heterogeneity (whose interpretation depends importantly on the number of studies) or the between-study variance as estimated in a random-effects meta-analysis (whose interpretation depends importantly on the measurement scale). The 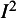 index was promoted in a *BMJ* paper by the same authors, along with Altman and Deeks, the following year.^3^^,^ ^4^ The notion of “proportion of total variance due to between-study variance” had previously been mentioned in passing by Takkouche and colleagues.^5^ Reporting of the 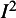 index in meta-analyses has increased year by year, extending far beyond the initial context for which it was initially proposed, and the measure has been widely implemented in different research fields.^6^^–^ ^10^ The index is now extremely widely used; the 2002 and 2003 papers frequently appear in lists of very highly cited papers.^11^^,^ ^12^ The journal *Nature* recently ranked the *BMJ* paper as the twentieth most cited of the twenty-first century.^13^

Although 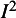 has been welcomed by some,^14^^,^ ^15^ its popularity has been accompanied by some common misconceptions and misinterpretations that hamper its usefulness in practice.^16^^,^ ^17^ When interpreted correctly as a measure of inconsistency rather than heterogeneity, the index has a useful place in the meta-analysis toolkit and its idea has been extended to other applications. In this paper, we provide a technical discussion of the 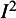 index, providing extensive references to related papers and our reflections from several years experience of working in the research synthesis field. We present different definitions of the 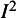 index, clarify its interpretation, address expressions of uncertainty, discuss extensions, and close with our suggestions on how the use and reporting of 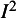 might be improved in future meta-analyses.

## Definitions of 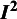


2

### Standard (Q-based) definition of 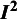


2.1

Perhaps the most familiar definition of the 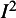 index is based on the 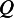 statistic, which is another commonly employed statistic used to examine the variability among the results included in the meta-analysis. In this approach, 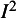 is defined as(1)

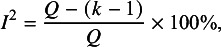

where 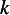 is the number of studies and 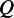 is the 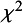 statistic proposed by Cochran and defined as follows^18^:(2)

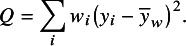



Here, 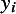 is the result from the 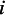 *^th^* study; 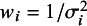 is the weight assigned to the result from the 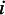 *^th^* study, with 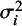 being the within-study variance reflecting the sampling error in 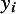 and typically assumed to be known in meta-analysis; and 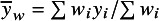 is an inverse-variance-weighted average of the study-specific estimates. Under the simplifying assumption that weights are known and identical for every study, the 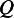 statistic follows a chi-squared distribution with 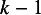 degrees of freedom.^19^ Although the 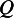 statistic has been widely used as a hypothesis test to detect heterogeneity among the individual results, it suffers from low statistical power in most meta-analytic applications and therefore can easily lead to misleading conclusions.^2^^,^ ^20^

Because the value of 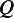 can be smaller than its degrees of freedom, the 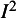 index defined in (1) can be negative. Therefore, in practice, 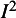 is customarily truncated at 0%, such that negative numbers (indicating less variation than would be expected by chance alone) are not allowed.

### Conceptual definition of 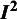


2.2

A broader definition of 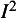 relates to the underlying parameters rather than to the test statistic 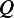 . Although the following definition appears to be parametric on the surface, it is not strictly parametric, as we shall discuss. We define a quantity 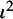 as(3)

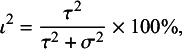

 where 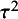 is the between-study variance parameter and 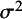 is a measure of within-study variance (i.e., the sampling error in estimation of 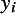 ). We use a Greek letter here to distinguish the parametric form, 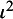 , from a statistic computed from data, for which we use 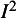 .

Numerous estimators of between-study variance, 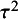 , have been described in the meta-analytic literature.^21^ A moment-based estimate, 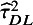 , introduced by DerSimonian and Laird, was popular for many years, although others are now considered preferable.^22^^,^ ^23^ The moment-based estimate is given by^24^


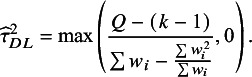



The within-study variance quantity 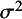 is a clearly defined parameter only if all studies have the same within-study variance. Otherwise, 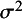 needs to be interpreted as a “typical” within-study variance across the studies included in the meta-analysis. Different choices are available for estimating such a typical variance, including the arithmetic mean, the median, and the geometric mean of the 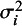 . HT proposed the quantity:(4)

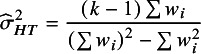

 with 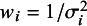 .^3^ Takkouche, Cadarso-Suárez, and Spiegelman (TCS) used^5^
(5)

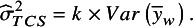



Since 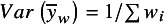 and 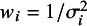 , it can be seen that 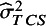 is the harmonic mean of the 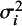 values.

Given that there are several options to estimate the between-study variance 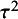 and several options to estimate a typical within-study variance 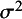 , numerous values of 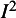 can be derived using Equation (3). The 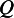 -based definition of 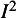 in Equation (1) corresponds to the choice of the DerSimonian and Laird estimator, 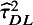 , and the use of 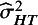 from Equation (4). We refer to this as the “method-of-moments” (MM) definition: (6)

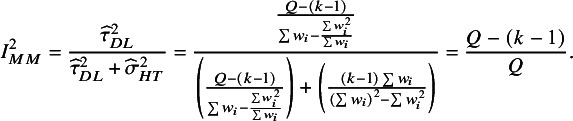



### Heuristic definition of 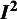


2.3

Noting the broad interpretation of 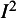 as the proportion of total variation in observed results that arises from between-study heterogeneity,^3^^,^ ^25^ a further option to define 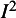 is as follows:(7)

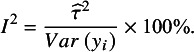



The denominator in (7) is just the sample variance of the study estimates. Under the usual additive heterogeneity model, rather than a multiplicative heterogeneity model,^26^ this can be regarded as conceptually equivalent to the sum of the between-study ( 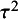 ) and within-study ( 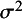 ) variance components in the denominator of Equation (3).^25^

### General definition of 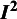


2.4

The conceptual definition presented in Equation (3) is analogous to using weighted sums of squares as follows:(8)

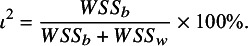



In this expression, 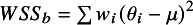 is the between-study weighted sum of squares and quantifies the dispersion of the effect parameters from each study ( 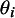 ) from the overall mean 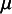 . The term 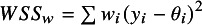 is the within-study weighted sum of squares, which accounts for the dispersion of the estimates ( 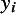 ) from the effect parameters 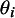 for each study.

We can verify the conceptual equivalence of (8) with (3) and (7) by noting that (9)

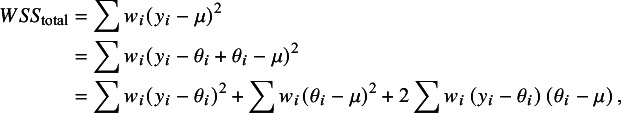

 where 
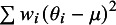
 corresponds to 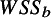 and 
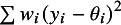
 corresponds to 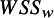 . The final term, 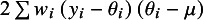 , represents residual covariation between the within- and between-study variance components. When within-study variances are uncorrelated with effect sizes, this covariation will be zero and the 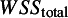 is equivalent to the sum of 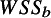 and 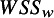 .

The general definition of 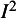 presented in Equation (8) provides a framework to specify the “true” value of the index ( 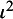 ) in simulation studies. We used this definition for the study presented in the next section.

## Illustration of the properties of different 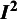 estimators

3

### 3.1 Application to two real data sets

Simulation studies indicate that alternatives to 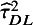 have better properties,^22^^,^ ^23^ so the question arises as to whether using an alternative estimator for 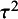 in (3) would be preferable for computing 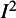 . In addition, since different options are available for estimating 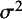 , it is not immediately clear which should be used. In a later section, we examine some of these issues in a simulation study. Here, we illustrate the impact that different choices can have through application to two examples, using the *metafor* R package for the calculations.^27^

The first example is a set of 13 clinical trials of Bacillus Calmette–Guerin (BCG) vaccine to prevent tuberculosis,^28^ using data presented by Berkey and colleagues.^29^ The effect measure for this example is the log risk ratio comparing the risk of tuberculosis in the vaccinated versus unvaccinated groups. In Table 1, we list different values of 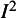 for different estimators of 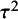 and 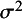 as well as using the simple sample variance of the 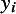 as the denominator (Equation (7)). A key point we seek to illustrate here is that 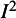 values range markedly, from a minimum of 47.4% to a maximum of 94.2%. The usual value, based on the DerSimonian–Laird estimator of 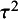 ( 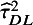 ) and the HT “typical variance” formula ( 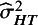 ), is 92.1%.

**Table 1:** Different values of 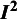 obtained using the different definitions, with different choices of estimators for 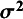 and 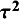 , applied to studies of BCG vaccine

		Choice for 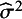					
		Arithmetic mean	Median	Geometric mean	HT	TCS	Proportion of total sample variance
Choice for 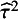	DL	66.9%	81.2%	82.8%	**92.1%**	93.5%	64.1%
	Hedges	68.3%	82.1%	83.6%	92.6%	93.9%	68.3%
	HS	59.9%	76.2%	78.0%	89.6%	91.5%	47.4%
	SJ	69.3%	82.9%	84.3%	92.9%	94.2%	71.8%
	ML	64.7%	79.7%	81.3%	91.4%	92.9%	58.2%
	REML	67.2%	81.4%	83.0%	92.2%	93.6%	65.1%
	EB/PM	67.5%	81.7%	83.2%	92.3%	93.7%	66.1%

*Note*: The bold entry is the “standard” choice ( 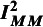 ) as introduced by Higgins and Thompson.

HT, Higgins and Thompson; TCS, Takkouche, Cadarso-Suárez, and Spiegelman; DL, DerSimonian–Laird; HS, Hunter–Schmidt; SJ, Sidik–Jonkman; MLE, maximum likelihood; REML, restricted maximum likelihood; EB, empirical Bayes; PM, Paule–Mandel.

The second example is a set of results from 19 studies examining how teachers’ expectations influence their pupils’ intelligence quotient (IQ) scores, a phenomenon sometimes labeled as the “Pygmalion effect” using data provided by Raudenbush (Table 2).^30^ The effect measure for this example is the standardized mean difference. Data were obtained from the metadata R package.^31^ Again, 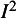 values range notably, from a minimum of 9.8% to a maximum of 76.1%, with the usual value using 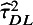 and 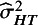 being 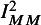 = 49.8%. The question then arises as to whether any of these variants is “more correct” than others, and we turn to this question in the following section by conducting a small simulation study.

**Table 2:** Different values of 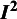 obtained using different definitions, with different choices of estimators for 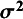 and 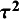 , applied to studies on the “Pygmalion effect”

		Choice for 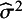					
		Arithmetic mean	Median	Geometric mean	HT	TCS	Proportion of total sample variance
Choice for 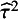	DL	34.9%	48.1%	42.7%	**49.8%**	50.6%	20.1%
	Hedges	62.4%	74.2%	69.8%	75.4%	76.1%	62.4%
	HS	31.6%	44.5%	39.2%	46.1%	47.0%	17.4%
	SJ	61.4%	73.4%	68.9%	74.7%	75.3%	59.9%
	ML	20.6%	31.0%	26.6%	32.4%	33.2%	9.8%
	REML	28.0%	40.3%	35.1%	41.9%	42.7%	14.6%
	EB/PM	51.7%	65.0%	59.8%	66.4%	67.2%	40.2%

*Note*: The bold entry is the “standard” choice ( 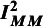 ) as introduced by Higgins and Thompson.

HT, Higgins and Thompson; TCS, Takkouche, Cadarso-Suárez, and Spiegelman; DL, DerSimonian–Laird; HS, Hunter–Schmidt; SJ, Sidik–Jonkman; MLE, maximum likelihood; REML, restricted maximum likelihood; EB, empirical Bayes; PM, Paule–Mandel.

### 3.2 A simulation study

#### 3.2.1 Simulation design

We conducted a small simulation study to examine the different approximations to the “typical” within-study variance in Equations (3) and (8) in the context of a very large meta-analysis (1,000 studies). For each meta-analysis, we drew 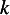 = 1,000 effect parameters 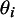 from a normal distribution with 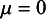 and 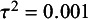 and then generated individual scores on a continuous outcome for two-arm studies from normal distributions with means 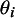 and 0 for the experimental and control arms, respectively, and with a between-individual variance of 1 for both arms. The effect measure for each study was Hedges’ *g*, that is, the standardized mean difference as presented by Hedges and Olkin.^32^ We assigned arm sizes of 300, 350, 400, 450, and 1,000 individuals, with each value being assigned to 200 studies in the meta-analysis, with equal arm sizes for the two arms within each study. Total study sizes were therefore 600, 700, 800, 900, or 2,000 individuals, and the corresponding within-study variances ( 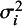 ) were 0.0067, 0.0057, 0.0050, 0.0044, or 0.0020.

We simulated 10,000 meta-analyses using R version 4.3.3.^33^ For each simulated meta-analysis, we computed the within-study effect estimate ( 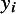 ) and resulting “true” 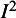 using a general weighted sums of squares approach (Equation (8)) based on known values of 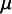 and 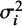 and simulated values of 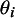 . We compared these true values with the values yielded by replacing 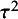 in Equation (3) with the variance of the effect parameters 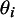 (to avoid selecting a specific estimator for 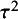 ) and using different estimators of 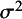 in Equation (3): arithmetic mean, median, HT (Equation (5)), and Takkouche and colleagues (Equation (6)). We also included the method using the total sampling variance of the observed effect estimates (Equation (7)).

#### 3.2.2 Simulation results

Table 3 shows the results of the simulation. Weighted sums of squares 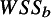 and 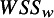 (see Equation (8)) yielded values of 0.001 and 0.004, respectively, the former corresponding to the preset known value of the between-study variance component and the latter defining the true value of the within-study variance component (this coincides with the harmonic mean of the vector of five preset 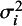 values), leading to a true parameter value of 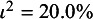 . Among the methods we compared, the formulae proposed by HT and by Takkouche and colleagues both yielded the correct average values of 0.004 for within-study variation. Conversely, using the median (0.005) or the arithmetic mean (0.005) led to the overestimation of within-study variation, resulting in an underestimation of 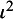 (by 3.3% and 2.7%, respectively). Lastly, the method using the total sampling variance of the observed effect estimates also underestimated the true value of 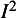 with an average value of 17.4%. Therefore, the different methods proposed to quantify a representative value of the within-study variation across studies, 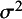 , showed some discrepancies that affected the estimation of 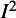 using Equation (3), with only the Takkouche and the HT methods performing close to the general formula presented in Equation (8). The lesson from our simulations is that only these two approaches should be considered (from those compared) for summarizing within-study variances across studies. Comparisons of these two methods in less favorable simulated scenarios can be found elsewhere.^34^^,^ ^35^ Our simulations involved large numbers of studies so as to ensure that the between-study variance was well estimated. Further simulation studies might elucidate preferred estimators of between-study variance, although we would expect findings to align with existing recommendations for the choice of between-study variance.^22^^,^ ^23^

**Table 3:** Results from the simulation study

Approach	Between studies	Within studies	*I*^2^
WSS	0.001	0.004	20.0%
Variances: HT	0.001	0.004	20.0%
Variances: TCS		0.004	20.0%
Variances: Med		0.005	16.7%
Variances: AM		0.005	17.3%
Total variance	0.006		17.4%

WSS, weighted sums of squares; HT, Higgins and Thompson; TCS, Takkouche, Cadarso-Suárez, and Spiegelman (harmonic mean); Med, median; AM, arithmetic mean.

## Expressing uncertainty in 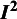


4

Values of the 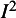 index can be highly volatile when based on a small number of studies.^36^ In many fields, very few studies tend to be included in meta-analyses (with a median of 3 having been reported in the health area^37^). HT originally derived formulae to calculate a confidence interval around 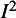 based on 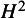 , another metric for quantifying inconsistency in meta-analysis sometimes known as Birge’s ratio.^3^ An alternative, preferable, confidence interval for 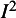 is based on a noncentral chi-squared distribution,^38^ used by Orsini.^39^ Hedges and Pigott note that this is the distribution of Q under a fixed-effects model, while under a random-effects model it has a gamma distribution.^38^ These results are used as the default in the *metan* macro for Stata.^40^

Confidence intervals for 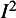 may also be derived from confidence intervals for 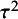 . Methods for these include the Q-profile method,^41^^,^ ^42^ a generalized Q-statistic method,^43^^,^ ^44^ and a profile-likelihood method,^45^^,^ ^46^ all of which are implemented in the metafor package for R.^27^^,^ ^33^ Additional approaches have been developed that do not require a known sampling distribution of the *Q*-statistic^47^^,^ ^48^ an assumption which has been deemed unrealistic in most applied situations.^19^ To the best of our knowledge, no simulation studies comparing all existing methods have been conducted to date, although an empirical comparison of the performance of some of them was recently conducted by Wang and colleagues.^49^

## Interpretation of 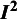


5

The 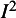 index is often interpreted as the amount of heterogeneity among studies, that is, as a measure of the variability across studies in true effect sizes underlying the effect estimates. Despite being widespread in the literature, this interpretation is incorrect. Numerous papers have explained this misinterpretation, and we refer the reader to several clear expositions.^16^^,^ ^17^^,^ ^50^^–^ ^52^ The 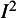 index measures the amount of heterogeneity *in relation to the sampling error associated with the effect estimates feeding into the meta-analysis*. As such, it is similar in interpretation to the intraclass correlation coefficient in clustered data. In fact, the letter 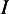 was originally chosen to represent “intraclass,” although we regard it as more constructive to consider the 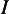 to stand for “inconsistency.”


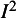
 may be interpreted as the proportion of variability in point estimates that is due to heterogeneity rather than sampling error. Informally, it can be considered to reflect the extent to which confidence intervals displayed in a forest plot do not overlap with each other. This is the rationale for the characterization of 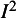 as a measure of *inconsistency*. If the results of the different studies are consistent with each other, their confidence intervals will have a high degree of overlap. If the results are inconsistent, there will be poor overlap in the confidence intervals. Note that the degree (or not) of overlap in confidence intervals is unrelated to the numbers on the effect measure axis. 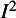 is invariant to a linear transformation of point estimates and confidence interval limits (in the metric used for the analysis, which is typically the logarithmic scale if the effect is measured on a ratio scale). Thus, 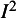 cannot be a measure of absolute heterogeneity, which is a quantity that is specific to the actual numbers on the axis.

A fundamental property of 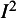 is that it will increase as the study effect estimates get more precise. Specifically, as studies get larger, confidence intervals around their effect estimates get narrower, and the overlap in confidence intervals across studies reduces, leading to larger values of 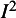 . This is apparent from Equation (3): For a given set of true effect sizes 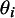 (and hence fixed 
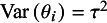
 ), the value of 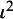 increases as the typical within-study variance, 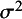 , decreases.^17^

A much lesser problem in the interpretation of the 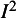 index is that it has a minor dependence on the number of studies ( 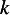 ) in the meta-analysis, as simulation studies have shown. Specifically, 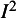 has been reported to yield biased estimates when the number of studies, 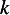 , is very small,^25^ similar to the pseudo- 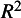 statistic, another ratio measure in meta-analysis that requires estimation of 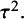 ^53^ Furthermore, the 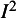 index is closely related to the 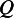 statistic, and hence, analogous to the low statistical power of the test based on the 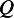 statistic, it has substantial uncertainty unless a large number of results is combined.^35^^,^ ^54^ Performance of both the *Q*-statistic and the 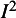 index has also been found to be affected by publication bias simulated by Augusteijn and colleagues, who additionally developed a web-based application to explore the potential impact of publication bias on the assessment of heterogeneity for a given meta-analytic data set.^55^

An important additional point worth noting here is that there are technical problems with 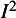 arising from the fact that the within-study variances are not known but estimated with error. These problems, which will be more important when study estimates have low precision, are well articulated by Hoaglin.^19^^,^ ^56^

### What is a large value of 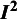 ?

5.1

It is tempting to seek ranges or thresholds for the interpretation of 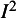 : What is a large amount of inconsistency? The original paper proposing the index proposed that “mild heterogeneity might account for less than 30 per cent of the variability in point estimates, and notable heterogeneity substantially more than 50 per cent.” The use of the word “heterogeneity” in these proposals, while correct, may have led readers to believe the descriptors “mild” and “notable” related to the amount of heterogeneity rather than the amount of inconsistency. This is unfortunate. The arguments were based informally on the connection between heterogeneity and inconsistency for situations in which the chi-squared test for heterogeneity achieves statistical significance (at a 5% level) for around 10–30 studies, with meta-analyses of randomized trials very much in mind.^3^ The benchmarks did not directly consider the absolute magnitude of heterogeneity, as measured by 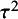 .

The most widely cited paper about 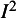 proposed “naive categorisation of values for 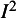 would not be appropriate for all circumstances, although we would tentatively assign adjectives of low, moderate, and high to 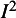 values of 25%, 50%, and 75%.”^4^ Again, these proposals were intended to apply only to randomized trials. Despite the caveats, these benchmarks have become widely (mis)used and applied to situations far beyond meta-analyses of trials.

In an attempt to curb the overzealous use of the thresholds, the *Cochrane Handbook for Systematic Reviews of Interventions* in 2008 proposed overlapping ranges, again intended for use only with randomized trials: 0% to 40% might not be important; 30% to 60% may represent moderate heterogeneity; 50% to 90% may represent substantial heterogeneity; 75% to 100% may represent considerable heterogeneity.^57^ These suggestions were accompanied by clear guidance that “the importance of the observed value of 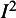 depends on (i) magnitude and direction of effects and (ii) strength of evidence for heterogeneity (e.g., *P*-value from the chi-squared test, or a confidence interval for 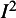 ),” advice which has again largely been ignored in practice.

Although these benchmarks can be helpful as a very rough guide, they should not be used to categorize meta-analysis data sets or to decide on whether, or how, to combine the results. We particularly discourage the use of thresholds for 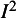 to decide between adopting fixed-effect(s) or random-effects meta-analysis models, because such decisions should be made on the basis of the question being asked rather than characteristics of the data.^58^ Empirical investigations of typical values of 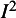 can provide some useful pointers as to how a particular meta-analysis data set compares with others in related areas. Rhodes and colleagues reanalyzed 9,895 meta-analysis data sets from Cochrane reviews and fitted distributions to them.^59^ For meta-analyses of trials with binary outcomes, the median value of 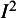 was 22% for log odds ratios, with interquartile range (IQR) 12% to 39%. For continuous outcomes meta-analysis, the median was 40% for standardized mean differences, with IQR 15% to 73%. Such a difference between binary and continuous outcomes has been noted by others.^60^ Differences have also been reported depending on the choice of effect measure within categorical^59^^,^ ^61^ and continuous^54^^,^ ^59^ outcomes, with, for example, inconsistency being higher for risk differences than for odds ratios or risk ratios.

Application of 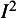 to results other than treatment effects from randomized trials can yield extreme results. An overview of 134 meta-analyses of prevalence studies found a median 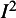 value of 96.9% (IQR: 90.5% to 98.7%).^62^ An overview of 138 reliability generalization meta-analyses found a median 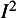 value of 93.2% (IQR: 88.8% to 96.4%) when integrating untransformed alpha coefficients, with similar results for the transformations typically used in this field.^63^ These very high values reflect the higher precision with which these quantities are estimated in relation to the underlying heterogeneity. For example, studies of prevalence can be very large, being based on large surveys or databases. In this situation, even trivially small differences in prevalence can lead to very large values of 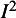 . The utility of the 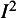 index is therefore questionable in such situations, although as a statistic it cannot be said to be misleading or wrong.

## Extensions and repurposing of 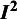


6

The popularity of the 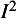 index has led to several extensions for application to specific types of meta-analysis. One of these is to meta-analyze models with covariates (known as moderator analysis or meta-regression). Here, part of the between-study variation is hoped to be explained by covariates, with some residual variation that cannot be accounted for by the model covariates. In this context, 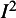 is interpreted as the proportion of the residual between-study variation (rather than the total variation) due to heterogeneity as opposed to random sampling error.^64^

Other extensions for more complex models include variants for multilevel meta-analytic models^65^ and multivariate meta-analysis,^66^^,^ ^67^ with the latter being applicable also in the context of network meta-analysis.^68^^,^ ^69^ Multivariate versions were initially based on normal–normal random-effects models and have been extended to binomial–normal random-effects models for use, for example, in meta-analysis of diagnostic test accuracy studies.^70^ Analogs to 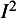 have additionally been proposed for individual participant data meta-analysis and cluster-randomized trials.^71^

Vo and colleagues extend the use of the 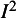 index to examine the impact of different sources of heterogeneity.^72^ They partition the heterogeneity variance, 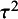 , into heterogeneity due to differences in participant characteristics (“case mix”) and heterogeneity due to other sources (largely methodological differences across the studies). They then propose versions of the 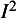 index for each of these components separately, expressing each as a proportion of the total variation in study estimates. Note that, in practice, the separation of these two sources of heterogeneity can only be achieved with individual participant data.^72^

The 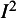 index has been repurposed for application to other problems. Montori and colleagues apply the 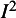 index to multiple waves of a survey, where at each wave an attempt is made to reach nonresponders to earlier waves.^8^ The 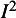 index calculated from answers to a survey question across multiple waves provides a measure of the inconsistency across waves due to response bias. Specifically, it describes the proportion of total variation in responses to the question, in successive waves, that is due to differences in the responders across waves rather than random variation within each wave. Finally, Bowden and colleagues adopted the 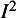 index for use in the field of Mendelian randomization to quantify the strength of violation of an assumption made in two-sample Mendelian randomization.^73^

## 7 Alternative indices of heterogeneity

Alongside the 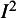 index, popular statistics for assessing heterogeneity in a meta-analysis are the Q-statistic and the between-study variance parameter. The latter is easier to interpret when reported as the standard deviation, 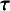 . This standard deviation may also be used to calculate prediction intervals around the overall effect estimate.^74^ Prediction intervals provide a range of plausible values for the true effect in a further primary study similar to those already in the meta-analysis. They directly portray the amount of heterogeneity observed in the meta-analysis on the metric used to present the meta-analytic results. The use and reporting of prediction intervals is now widely advocated in the meta-analytic literature.^74^^–^ ^80^

Assessment of heterogeneity is a key aspect of any meta-analysis, so it is not surprising that several alternatives to these indices have been proposed.^81^ Lin and colleagues^82^ developed two indices intended for meta-analyses including outlying studies and reported a robust performance under such scenarios.^83^ Crippa and colleagues proposed another scale-invariant measure, which, unlike the 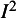 index, requires neither quantification of a typical value of the within-study variance nor an assumption of homogeneity of these quantities.^84^ The proposed index quantifies the between-study heterogeneity relative to the variance of the summary random-effects estimate. A disadvantage of this measure is the potential for bias, especially with a small number of studies.^81^ The coefficient of variation, calculated as the ratio between 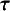 and either the fixed-effects^5^ or random-effects^85^ overall estimate, has been proposed as a complementary measure of heterogeneity, although it can be expected to yield very large values when the effect estimate is small, which hampers interpretation.^86^

Last, we note that HT originally proposed the 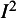 index along with two other quantities.^3^ The first of these was the H index or Birge’s ratio, closely related to the standard (Q-based) definition of 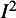 , and with a modified version subsequently proposed.^35^ The second was the 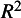 index, a ratio of the variances of the random-effects and the fixed-effects overall estimates in the meta-analysis, subsequently rebranded as the diamond ratio.^87^

## 8 Discussion

Papers proposing the 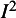 index for meta-analysis are among the most highly cited in the medical research literature, with the original papers in *Statistics in Medicine* and *BMJ* having been cited in excess of 35,000 and 60,000 times, respectively. In this paper, we have reviewed the properties and interpretations of the 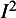 index. We overviewed different ways of defining the statistic and illustrated how different definitions can lead to different values.

When a fixed-effects model is assumed, there is no estimation of the between-study variance, 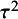 , and hence, we regard the standard (Q-based) definition to be a natural choice to compute 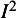 . Furthermore, if both fixed- and random-effects meta-analyses are planned, then the Q-based definition has the advantage that it will yield the same result for both statistical models. In the context of a random-effects meta-analysis, other definitions for 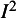 are serious contenders. A particular problem is that the closest version to a parametric interpretation of the 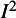 index (our conceptual definition) involves the notion of a “typical” within-study variance term, which is a poorly defined quantity. As a result, we propose a general definition of 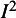 based on weighted sums of squares and use this as a basis for a small simulation study. We suggest that this new definition may have a useful role in a range of future simulation studies investigating methods for estimating heterogeneity parameters or mean meta-analytic effects.

We believe the index plays a very valuable role in the meta-analysis toolkit, although we are concerned at the widespread misunderstanding and misinterpretation of it in practice. Common mistakes are to interpret the 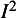 index as a measure of the absolute amount of heterogeneity across studies and to apply inappropriate thresholds for interpreting values of the index. It is important to remember that the 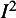 index is a relative measure of inconsistency in results rather than an absolute measure of heterogeneity in effects. It intentionally depends on the precisions (or within-study variances) of the study estimates contributing to the meta-analysis. One consequence of this is that it cannot be used to measure the amount of heterogeneity; its value is wholly uninformative about variation in effect sizes on the scale used to measure the effect. We suspect that many users of the 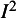 index have little appreciation of the actual degree to which the effects in their meta-analysis vary from study to study, which may lead them to inappropriate interpretations of their results. A second consequence is that, in some situations (mainly when studies are large), the value of 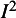 can be expected to be large. This does not mean that use of the 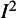 index is wrong, merely that it may not be the most helpful measure. To make the most of the imperfections in 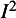 , we offer a series of recommendations for its appropriate use (see Box).

Box. Recommendations for use of the 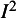 index in meta-analyses.

1. Ensure 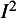 is appropriately interpreted as a *relative* measure (of inconsistency) and not an *absolute* measure (of heterogeneity).
2. Recognize that, in many situations, large studies will naturally lead to large values of 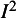 . This is not a problem; it is inherent in the purpose of 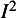 as a measure of inconsistency of results rather than heterogeneity of effects.
3. Accompany 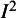 with some measure to convey the strength of evidence for whether heterogeneity is truly present. This could be, for example, a confidence interval for 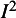 or an exact P value from the Q test for heterogeneity.
4. Avoid strong reliance on thresholds for interpreting 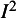 . Any thresholds that are used should be appropriate to the type of study, noting that commonly cited thresholds were derived specifically for clinical trials.
5. Be aware that 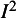 may naturally be large for meta-analyses of single-group studies, particularly when true values can be variable in relation to the sampling errors involved in estimating them. This is particularly true, for example, in meta-analyses of prevalence estimates or sensitivity/specificity estimates in test accuracy studies.
6. To describe the actual amount of heterogeneity across studies, present the between-study standard deviation in effects ( 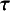 ) rather than 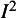 . Note that this standard deviation is usefully portrayed using a prediction interval in the context of a random-effects analysis.

## Data Availability

The R code used for the examples and simulation can be found at https://osf.io/yp7u4/.
